# Monitoring neurocognitive functioning in childhood cancer survivors: evaluation of CogState computerized assessment and the Behavior Rating Inventory of Executive Function (BRIEF)

**DOI:** 10.1186/s40359-019-0302-3

**Published:** 2019-05-02

**Authors:** Lyn M. Balsamo, Hannah-Rose Mitchell, Wilhelmenia Ross, Catherine Metayer, Kristina K. Hardy, Nina S. Kadan-Lottick

**Affiliations:** 1 Yale University School of Medicine, 15 PO Box 208064, 16 333 Cedar Street, LMP-2073 (for courier mail), 17, New Haven, CT 06520-8064 USA; 2 University of Miami, Coral Gables, FL USA; 3 School of Public Health, University of California, Berkeley, California USA; 4 Center for Neuroscience and Behavioral Medicine, Neuropsychology Division, Children’s National Medical Center, Washington, DC, USA; 5 Department of Psychiatry and Behavioral Science, George Washington University School of Medicine, Washington, DC, USA; 6 Yale Cancer Center, New Haven, CT USA

**Keywords:** Late effects, Neurocognitive, Neuropsychological evaluation, Computerized assessment, Survivorship

## Abstract

**Background:**

Many childhood cancer survivors develop neurocognitive impairment, negatively affecting education and psychosocial functioning. Recommended comprehensive neuropsychological testing can be time- and cost- intensive for both institutions and patients and their families. It is important to find quick and easily administered surveillance measures to identify those in need of evaluation.

**Methods:**

We evaluated, individually and in combination, the sensitivity and specificity of the 1) Behavior Rating Inventory of Executive Functioning-Metacognition Index (BRIEF-MCI), and 2) CogState Composite Index (computerized assessment of cognition) in identifying below grade-level performance on state-administered tests of reading and mathematics among childhood cancer survivors.

**Results:**

The 45 participants (39% female) were a mean age of 7.1 ± 4.4 years at diagnosis, 14.0 ± 3.0 at evaluation, with a history of leukemia (58%), lymphoma (9%), central nervous system tumors (20%), and other tumors (13%). Impairment on the BRIEF-MCI was associated with low sensitivity (26% reading, 41% mathematics) but stronger specificity (88% reading, 96% mathematics). We found similar associations for the CogState Composite Index with sensitivity of 26% for reading and 29% for mathematics and specificity of 92% for both reading and mathematics. Combining the two measures did not improve sensitivity appreciably (47% reading, 59% mathematics) while reducing specificity (84% reading, 88% mathematics).

**Conclusions:**

While individuals identified from the BRIEF-MCI or CogState Composite would likely benefit from a full neuropsychological evaluation given the strong specificity, use of these measures as screening tools is limited. With poor sensitivity, they do not identify many patients with academic difficulties and in need of a full neuropsychological evaluation. Continued effort is required to find screening measures that have both strong sensitivity and specificity.

## Background

More than 80% of children and adolescents diagnosed with cancer survive their disease. [1] Curative therapies are often neurotoxic and can be associated with neurocognitive difficulties. [2] The Children’s Oncology Group (COG) Long-Term Follow-Up Guidelines recommend routine surveillance for any pediatric cancer survivor at high risk for neurocognitive impairment. [3] [4, 5] [6] Unfortunately, traditional neuropsychological assessment is often time- and cost-intensive for patients and their families. Insurance reimbursement for evaluations is not uniform, even within the United States. [7] Further, evaluations typically require expertise in psychological testing that is not readily available at all pediatric cancer treatment centers. [8] In this context, comprehensive neurocognitive assessment for every child is ideal, but may not be a feasible goal. Therefore, efficient and accessible surveillance measures to identify children requiring comprehensive assessment are important to standard survivorship care. [9]

To achieve a better balance between needs, patient costs, and available resources, Hardy, Olson [10] recommend a tiered, prevention-based approach to neuropsychological assessment of children at risk for neurocognitive problems due to their medical conditions. The authors suggest universal monitoring at the first level of assessment, consisting of batteries that are short (completed in less than 30 min), psychometrically sound, and comprised entirely of computer-based tasks or parent-report instruments. [10] Among pediatric leukemia and brain tumor survivors, parent-report measures have demonstrated good specificity, but showed low sensitivity in identifying those with neurocognitive or psychosocial problems or those demonstrating real-life difficulties, e.g., receiving special education services. [11] [12] In contrast, Bull, Liossi [13] showed moderate to high sensitivity in using a combination of parent-, self-, and teacher-completed reports to identify those brain tumor survivors with below average IQ. These patients, however, had a particularly high prevalence of poor neurocognitive outcomes, and it is unclear if these findings generalize to survivors with a range of less severe neuropsychological difficulties.

Computerized testing is poised to be an excellent monitoring tool given its efficiency, reduced practice effects that allow more frequent evaluation and lower level of required clinical expertise for administration. In comparison to traditional neuropsychological testing, computer batteries are considerably shorter in duration, e.g., minutes versus hours, and can be more standardized in delivery. For tests of attention and processing speed, a multitude of precisely measured data can be acquired in short periods of time as stimuli can be presented rapidly and responses recorded to the millisecond, also increasing the sensitivity of the measure. Further, the data are computer-scored, thus reducing error, and administration does not usually require a doctoral-level clinician but a technician familiar with the system. [14] Computerized tests that are quick to deliver, contain multiple forms, and do not require rule learning can minimize practice effects. Some computer platforms, like CogState, were designed with the goal of repeated assessment, to detect accurately change over time and have been demonstrated to effectively minimize practice effects. [15–17] Previous studies of CogState in HIV-infected, schizophrenia, and multiple sclerosis patient groups have established its validity in children and adults, producing results comparable to traditional neuropsychological measures. [18, 19] It is currently being used in several COG trials at over 150 sites to measure the neurocognitive effects in children undergoing treatment for high-risk acute lymphoblastic leukemia (ALL) and acute promyelocytic leukemia (COG AALL1131 and AAML1331).

The validity of monitoring batteries in pediatric oncology has typically been measured by assessing their association with performance on other traditional neuropsychological tests; however, these tests may not capture well real-world functioning. [20, 21] Measures of academic performance are clinically meaningful, but infrequently utilized because of difficulty in obtaining results. [22] Further, many standardized assessments compare students’ performance to one another, rather than to an a priori established benchmark that indicates whether or not a student is meeting educational standards from a particular geographic area. Using a criterion-based measure at the state-level should have good ecological validity and best assess achievement in the child’s natural environment.

Childhood cancer survivors treated with chemotherapy, neurosurgery, or CNS-directed therapies are at varied risk for neurocognitive problems. [23–26] We intend to test the hypothesis that at least one of three methods: 1) Computerized assessment, CogState; 2) Questionnaire, Behavior Rating Inventory of Executive Function (BRIEF), Parent Form or Adult Version; and/or 3) Combined questionnaire and computerized assessment will be associated with reading and mathematics performance on state-administered criterion-referenced tests of reading and mathematics with adequate sensitivity and specificity to be used as neurocognitive monitoring tools. An exploratory aim of this study is to determine the cut-off scores that yield the best sensitivity and specificity.

## Methods

### Participants

Patients ≥8 years of age at evaluation and ≤ 18 years at cancer diagnosis were eligible for participation if they were English-speaking and treated with chemotherapy, cranial radiation, and/or neurosurgery. Additional eligibility criteria included ≥2 years elapsed from diagnosis; no pre-cancer diagnosis of learning disabilities, developmental conditions, and/or Attention-Deficit/Hyperactivity Disorder; and available results from state-administered achievement testing completed after the cancer diagnosis and within 5 years of participation in the present study. Between October 2012 through July 2013, research assistants enrolled 45 patients who arrived at the outpatient clinic for a routine visit (not for chemotherapy or acute illness). Signed informed consent was obtained from all patients or their parents as appropriate for age. Participants received a $20 honorarium. All study procedures were approved by the ethics review board at Yale University.

### Measures

*A demographic questionnaire* with items about race and maternal education was completed by parents of patients under age 18 or adult patients.

The *BRIEF-Parent Form* or *BRIEF-Adult Version* was completed by patients under age 18 or adult patients, respectively. [27] The BRIEF is validated for adults and parent proxy to yield eight scales from 75 to 86 items. These measures have high internal consistency (α’s = .80–.98) and test-retest reliability (*r*s = .82). [27] The questionnaire takes approximately 10 to 15 min to complete. The Metacognition Index, a composite scale assessing the ability to initiate activity, sustain working memory, organize materials, monitor, and plan/organize was used in the analysis. The authors report that T-scores > 65 (i.e., 1.5 standard deviation above the mean) indicate clinically significant symptomology, a cutoff which was adopted to define a patient “at-risk” in this study. [27]

Trained research assistants administered three *CogState* (version 7) tasks which takes approximately 10 to 15 min to complete in total: Detection, a test of processing speed; Identification, a test of attention; and One Back, a test of working memory. These tests assess domains of cognition typically affected by cancer treatment [28–30] and was adapted from the battery used in the multi-site COG study for children with high-risk ALL (COG AALL1131). [31] The CogState Composite was calculated by converting the average raw score to an age-referenced z-score using normative data provided by CogState. A Composite Score of z < − 1 (i.e., 1 standard deviation below the mean) was identified as performance in the “at-risk” range as determined by exploratory analysis (please refer to the Analysis and Results sections for additional information).

Results from the reading and mathematics portions of the *Connecticut Mastery Test* (administered to all children in grades 3 through 8) or the *Connecticut Academic Performance Test* (administered to all children in grade 10) were used as outcomes. These are criterion-referenced measures of academic achievement administered by the State of Connecticut’s Department of Education to all students attending public schools between 1985 and 2015. This was the only academic measure universally administered to all students in the state at this time. Students’ performance is evaluated against pre-determined achievement standards. Individual scores fall into 1 of 5 categories: Advanced, Goal, Proficient, Basic, and Below Basic. The latter 3 categories are considered to fall within the Below Goal range. For the purpose of this study, these guidelines were adopted and each participant’s performance on reading and mathematics was categorized dichotomously as meeting (Goal, Advanced) or not meeting (Proficient, Basic, Below Basic) the state-defined benchmark. [32]

### Statistical analysis

Descriptive statistics were calculated for the participant characteristics, performance on CogState, and results of the BRIEF. We calculated sensitivity, specificity, positive predictive power (PPV), and negative predictive power (NPV) statistics for scores in the at-risk range (see definitions above) for the BRIEF Metacognition Index (MCI) and the CogState Composite score separately and then in combination (i.e. impairment by either or both tools) for the outcomes of “meet” or “did not meet” reading and mathematics benchmarks from academic testing. The cutoff used for CogState was determined thorough exploratory analysis. We calculated sensitivity and specificity statistics using cutoffs corresponding to 1, 1.5, and 2 standard deviation from the mean CogState score for the academic outcomes. We selected the CogState cutoff that provided the best associations with the academic outcomes for the main analysis. All analyses were conducted in SAS version 9.4.

## Results

### Participants

One-hundred-eleven patients were approached as part of a larger study. There was no statistical difference in age, race/ethnicity, gender or diagnosis between those participants that enrolled and the 5 that declined to participate. Among the 45 participants that had available state achievement testing and thus met eligibility criteria for this study (Table 1), the mean age at evaluation was 14.0 years (SD = 3.0; range: 8.6–20.8) with a mean age at diagnosis of 7.1 years (SD = 4.4; range: 0.2–14.1). Four participants were 18 years or older. Seventy-one percent of participants were male and 51% reported their race/ethnicity as non-Hispanic white. Diagnoses included leukemia (57.8%), lymphoma (8.9%), central nervous system (CNS) neoplasm (20.0%) and other solid tumors (13.3%). Parent- or self-report of executive functioning, as well as scores on the performance-based computerized tasks fell within the average range (Table 2). State achievement testing was completed a median of 0.73 years (range − 0.32 - 4.2) before completion of CogState. Median time elapsed between diagnosis date and school testing was 5.7 years (range 0.78–13.86). Two patients completed academic testing within 1 year of diagnosis.

**Table 1:** Participant Characteristics (Total *N* = 45)

	Counts (%) or Means (SD, range)
Age at diagnosis (years)	7.1 (4.4, 0.2–14.1)
Age at evaluation (years)	14.0 (3.0, 8.6–20.8)
Time elapsed since diagnosis (years)	6.9 (3.5, 2.2–16.0)
Sex	
Male	32 (71.1)
Female	13 (28.9)
Maternal education:^a^	
High school or less	12 (27.3)
Training after high school/ some college College grad or post-grad training	11 (25.0) 21 (47.7)
Diagnosis:	
Leukemia	26 (57.8)
Lymphoma	4 (8.9)
Central nervous system neoplasm	9 (20.0)
Other^b^	6 (13.3)
Treatment:	
Intrathecal chemotherapy	27 (61.3)
Cranial radiation, including total body	10 (22.2)
Central nervous system surgery	8 (17.8)
Stem cell transplant	5 (11.1)

^a^Category does not sum to 45 due to unanswered questions; ^b^These included rhabdomyosarcoma [2], retinoblastoma [1], germinoma [1], Langerhans cell histiocytosis [1], neuroblastoma [1]

**Table 2:** Peformance on neurocognitive and academic measures

Measure	Mean (SD, range)	Percent patients in at risk^d^ range
CogState^a^		
Detection	102.9 (15.2, 54.5–119.5)	–
Identification	104.6 (11.5, 75.7–123.3)	–
One-Back	95.6 (12.0, 74.1–118.5)	–
CogState Composite^b^	0.3 (1.1, −2.8-1.8)	16
BRIEF Metacognition Index^c^	54.3 (12.8, 30.0–83.0)	18
CT Mastery Test Reading	–	42
CT Mastery Test Mathematics	–	40

^a^Standard scale score, Mean = 100, SD = 15; ^b^Z-score, Mean = 0, SD = 1; ^c^T-score, Mean = 50, SD = 10; ^d^Cutoff 1 SD below the mean (CogState) or 1.5 SD above the mean (BRIEF)

### Associations between monitoring measures and academic outcomes

At risk scores on the BRIEF-MCI were associated with poor sensitivity for below-grade level reading (26%) and mathematics (41%) performance (Table 3). In contrast, at-risk results on the BRIEF-MCI was associated with adequate to good specificity for below-grade level reading (88%) and mathematics (96%) performance. Positive predictive value (PPV) and negative predictive value (NPV) were 63 and 61% respectively for below-grade level reading and improved to 88 and 71% respectively for below-grade level mathematics.

**Table 3:** Sensitivity, specificity, positive predictive power, and negative predictive power associated with using impairment on neurocognitive measures to predict rates of below grade level academic performance

	Reading below grade level				Mathematics below grade level			
Neurocognitive Measure, Cutoff	Sensitivity, % (95% CI)	Specificity, % (95% CI)	Positive Predictive Value, % (95% CI)	Negative Predictive Value, % (95% CI)	Sensitivity, % (95% CI)	Specificity, % (95% CI)	Positive Predictive Value, % (95% CI)	Negative Predictive Value, % (95% CI)
BRIEF-MCI, T > 65	26 (7–46)	88 (75–100)	63 (29–96)	61 (45–77)	41 (18–65)	96 (88–100)	88 (65–100)	71 (55–86)
CogState Composite, z < − 1	26 (7–46)	92 (81–100)	71 (38–100)	62 (47–78)	29 (8–51)	92 (81–100)	71 (38–100)	66 (38–100)
BRIEF-MCI, T > 65 *or* CogState Composite, z < −1	47 (25–70)	84 (70–98)	69 (44–94)	68 (51–84)	59 (35–82)	88 (75–100)	77 (54–100)	76 (60–91)

*BRIEF-MCI* = Behavior Rating Inventory of Executive Function, Metacognition Index

At-risk scores on the CogState Composite score was associated with low sensitivity for below-grade level reading (26%) and mathematics (29%) performance. It yielded strong specificity for reading (92%) and mathematics (92%) outcomes. PPV is 71% for both reading and math performance. NPV is 62 and 66% for reading and math, respectively.

With the same criteria as used previously, at risk results on *either* the BRIEF-MCI or CogState Composite or *both* was associated with low sensitivity for below grade level reading (47%) and mathematics (59%). At risk results on at least one of these measures was associated with better specificity for below grade level reading (84%) and mathematics (88%). But similar to using either measure alone, PPV was 69 and 77% for reading and math respectively.

In the analysis presented above, the CogState cutoff of z < − 1 was used to identify participants in the at-risk range because exploratory analysis indicated that the cutoff produced the best sensitivity and specificity, relative to cutoffs of 1.5 and 2 SD from the mean score. For example, when using a cutoff of z < − 1.5 (1 SD below the mean) for the CogState Composite, sensitivity was reduced to 18% (from 26%) and specificity was improved to 96% (from 92%) for reading outcomes. For the combined analysis using a CogState Composite cutoff of z < − 1.5 with the BRIEF-MCI cutoff of T > 65 yielded slightly improved specificity for reading (88% compared to 84%) and mathematics (92% compared to 88%) outcomes.

## Discussion

We aimed to determine if three potential universal monitoring plans using short questionnaires and/or computer assessments would identify those pediatric cancer survivors demonstrating academic weaknesses on state-administered assessments. The results indicate that impairment on the BRIEF-parent or adult-version and CogState computerized assessment demonstrated high specificity, but low sensitivity in classifying those individuals who did not meet academic benchmarks. Combining these measures did not improve sensitivity significantly and mildly reduced specificity. That is, at-risk results on either or both screening measures were not more highly associated with academic impairment.

This study is unique in utilizing a uniformly delivered criterion-referenced standard of educational attainment used within the State of Connecticut. In part, these data are used by school systems to identify those children who need additional support and intervention. As such they are a meaningful indicator of a student’s success in the subject areas of reading and mathematics. Our data indicate that participants who show at-risk results on either monitoring measure are likely to demonstrate deficiencies in academic achievement. These measures can appropriate resources to individuals in need of comprehensive neurocognitive assessment. However, it is important to note that with poor sensitivity and low negative predictive value (NPV) the BRIEF-MCI and CogState Composite computerized assessment will not detect many pediatric cancer survivors that could benefit from comprehensive evaluation. Thus, if a patient were not identified by the BRIEF-MCI or CogState Composite, it would be incorrect to assume that they did not need additional testing. In terms of identifying children at risk for neurocognitive weaknesses, an ideal screening tool would not miss as many children in need of assessment.

These results are consistent with two other studies that have investigated the use of parent-report measures as surveillance tools for identification of pediatric leukemia and brain tumor survivors with neurocognitive or learning problems [11, 12]. As in the current study, these measures had good specificity but were not sensitive enough to identify survivors with negative outcomes. Specifically, among pediatric leukemia and brain tumor survivors, screening for attention problems using parent-report measures (e.g., Conners Parent Rating Scale and the Child Behavior Checklist) demonstrated good specificity but showed low sensitivity in identifying those with either lower IQ or diminished working memory or information processing speed. [11] Similarly, in another study parent-completed BRIEF questionnaires demonstrated good specificity, but results were not sensitive enough to identify those leukemia survivors demonstrating real-life difficulties in the form of receiving special education services or problems with attention. [12]

These results indicate that it will be important to identify other monitoring tools with good sensitivity to identify those patients with neurocognitive difficulties. There is ongoing work to create and validate standardized computerized assessments of cognition, given their portability, reduced administration time, and ease of administration. Computerized tests, including CogState, require a computer, internet access, and qualified staff (several hours of training). There is a cost to CogState (https://www.cogstate.com), like many other computer platforms; however, this is substantially less than the cost of a neuropsychological evaluation or the time of a neuropsychologist. Psychological expertise is not required to administer these tests, but interpretation of the data should be completed by a specialist. Overall, however, there is potentially a savings in professional time and cost that can be advantageous in smaller institutions with less resources. [33–35] Other computerized batteries include the NIH toolbox for the Assessment of Neurological and Behavioral Function, [35] which is being utilized in both cancer [36] and non-cancer populations. [37] Similarly, numerous other computerized batteries, such as the Cambridge Neuropsychological Test Automated Battery (CANTAB) [38], Comprehensive Instrument for Evaluating Mild Traumatic Brain Injury (CIEMTBI) [33], and Immediate Post-Concussion Assessment and Cognitive Testing (ImPACT) [39] are being used to test cognition quickly and reliably among those with traumatic brain injury, CNS tumor, and mental health disorders.

A comprehensive history gathered through clinical interview and/or a review of the medical record can increase the sensitivity of surveillance [40]. A thorough history may include questions of patients, family, and/or teachers about grades earned, classroom behaviors, and performance. It is noted, however, that there is often low concordance between teacher- and parent-report and performance-based measures among pediatric cancer survivors. [41, 42] Brain tumor survivors are also more likely to under-report problems. [43] This is concerning as cognitive problems may go undetected in this high-risk population if we rely solely on self- and/or proxy-report to prompt comprehensive testing, which ultimately can delay intervention. Longer batteries that combine traditional neuropsychological performance-based measures with parent-proxy [44] have shown good ability to identify those with academic problems on nationally normed tests of achievement. However, these methods may not be standardized across settings or interviewers and may require more time and expertise than is available.

This study should be considered in the context of certain limitations. The sample size is small and represents participants from only one tertiary care center. Moreover, because a state-specific assessment was used, these results are most relevant to the state and limit generalizability. However, it may also be viewed as a strength of the study. Because academic performance varies among states, the best criteria for evaluating academic achievement would derive from expectations specific to the childhood cancer survivor’s educational environment. Additionally, this study only examined cancer survivors who were at least 2 years from diagnosis rather than earlier in the therapy period so generalizations to these patients cannot be made. It should be noted that 2 participants completed academic testing within 1 year of diagnosis, which would potentially miss neurocognitive effects that emerge later. Because these 2 participants showed agreement between academic testing and the later completed study assessments, it would not have appeared to affect the data. Academic achievement is increasingly recognized as an important clinical outcome [45]; however, the association between performance on neurocognitive domains such as attention and working memory and academic achievement is not as strong as a measure of overall ability such as IQ [46]. It may be promising to examine other downstream measures that are associated with neurocognitive issues, such as educational attainment; however, the length of follow up did not allow for that in this particular study [47]. Additionally, most participants (92%) exhibited adequate abilities in the areas tested, e.g., executive functioning, attention, working memory, and processing speed so the relatively small sample size of the present study may result in poor sensitivity. These potential monitoring tools may have stronger specificity in patients at highest risk for neurocognitive deficits (e.g., brain tumor patients), although these are individuals who may be the most appropriate for comprehensive evaluation based on their diagnosis and treatment alone.

## Conclusion

With increasing numbers of pediatric cancer survivors, it will be important to have an easily and quickly administered monitoring tool, particularly in resource-limited communities, to identify those patients in need of comprehensive neurocognitive assessment. While individuals identified from the BRIEF-MCI or CogState Composite would likely benefit from a full neuropsychological evaluation given the strong specificity, use of these measures as screening tools is limited. They do not identify many patients with academic difficulties and in need of a full neuropsychological evaluation. Continued effort is required to find screening measures that have both strong sensitivity and specificity.

## Abbreviation

BRIEF-MCI: Behavior Rating Inventory of Executive Function – Metacognition Index
COG: Children’s Oncology Group
